# Pharmaceutical Compounds in Aquatic Environments—Occurrence, Fate and Bioremediation Prospective

**DOI:** 10.3390/toxics9100257

**Published:** 2021-10-09

**Authors:** Joana P. Fernandes, C. Marisa R. Almeida, Maria A. Salgado, Maria F. Carvalho, Ana P. Mucha

**Affiliations:** 1 CIIMAR—Interdisciplinary Centre of Marine and Environmental Research, University of Porto, Terminal de Cruzeiros do Porto de Leixões, Avenida General Norton de Matos s/n, 4450-208 Matosinhos, Portugal; calmeida@ciimar.up.pt (C.M.R.A.); up412008@g.uporto.pt (M.A.S.); mcarvalho@ciimar.up.pt (M.F.C.); amucha@ciimar.up.pt (A.P.M.); 2 School of Medicine and Biomedical Sciences (ICBAS), University of Porto, Rua de Jorge Viterbo Ferreira 228, 4050-313 Porto, Portugal; 3 Faculty of Sciences, University of Porto, Rua do Campo Alegre 790, 4150-171 Porto, Portugal

**Keywords:** pharmaceuticals, microorganisms, bioremediation

## Abstract

Various contaminants of emerging concern (CECs) have been detected in different ecosystems, posing a threat to living organisms and the environment. Pharmaceuticals are among the many CECs that enter the environment through different pathways, with wastewater treatment plants being the main input of these pollutants. Several technologies for the removal of these pollutants have been developed through the years, but there is still a lack of sustainable technologies suitable for being applied in natural environments. In this regard, solutions based on natural biological processes are attractive for the recovery of contaminated environments. Bioremediation is one of these natural-based solutions and takes advantage of the capacity of microorganisms to degrade different organic pollutants. Degradation of pollutants by native microorganisms is already known to be an important detoxification mechanism that is involved in natural attenuation processes that occur in the environment. Thus, bioremediation technologies based on the selection of natural degrading bacteria seem to be a promising clean-up technology suitable for application in natural environments. In this review, an overview of the occurrence and fate of pharmaceuticals is carried out, in which bioremediation tools are explored for the removal of these pollutants from impacted environments.

## 1. Introduction

The presence of so-called contaminants of emerging concern (CECs) in the environment has been reported worldwide in the past years, mostly due to development in industrial and medical sectors. Although the presence of CECs in the environment has been occurring for decades, only in the past 10–15 years, analytical methods with the capacity to detect these pollutants at environmentally relevant concentrations have been developed [1,2,3].

CECs comprise different contaminants, such as pharmaceuticals, engineered nanomaterials, illicit drugs, synthetic musks, food additives, phthalates, hormones, steroids, industrial compounds/by-products and personal care and veterinary products [4,5,6,7]. At present, water quality framework regulations for these compounds do not exist, despite their potential threat to the environment and public health [7,8].

Until recently, monitoring or public reports of the presence of CECs in freshwaters or wastewaters were not required [9]. In 2013, Directive 2013/39/EU [10] was implemented as an amendment to Directives 2000/60/EC and 2008/105/EC, encompassing CECs as priority substances in the field of water policy, in an effort to regulate aquatic environmental contamination by these compounds. To achieve this goal, new high-quality monitoring and prioritization measures were implemented, according to article 16 of Directive 2000/60/EC [11,12]. A watchlist, containing several contaminants of emerging concern, was created to register the monitoring data and establish the risk that these selected contaminants may pose to the environment [13]. The first Watch List was published by Decision 2015/495/EU [13] and revised by Decision 2018/840/EU [14]. In 2020, a new, revised version was published in Decision 2020/1161/EU, including 18 CECs that should be monitored to gather information for further evaluation [15].

The risks associated with the presence of CECs in the environment and public health have been attracting more attention. Many CECs can be toxic, persistent and non-biodegradable [16], causing difficulties in their management. However, even the non-persistent compounds can cause negative effects in aquatic systems and organisms [17] if their continuous input in the environment exceeds their natural attenuation and transformation [18,19]. Therefore, besides monitoring and controlling CEC emissions, their removal from the impacted environments is necessary.

Pharmaceuticals, a well-known class of CECs, have been essential for the sustainability and maintenance of human health, ensuring life quality [20] and illness relief. Nonetheless, their extensive consumption has led to their presence in the environment, threatening living organisms [21]. The uncertainty regarding pharmaceuticals effects on different non-target organisms have been raising concern among the scientific community. Thus, there is an urgent need for the development of suitable technologies for recovering/remediating environments impacted by the presence of these pollutants, in addition to other reducing or preventive strategies.

**Figure 1:**
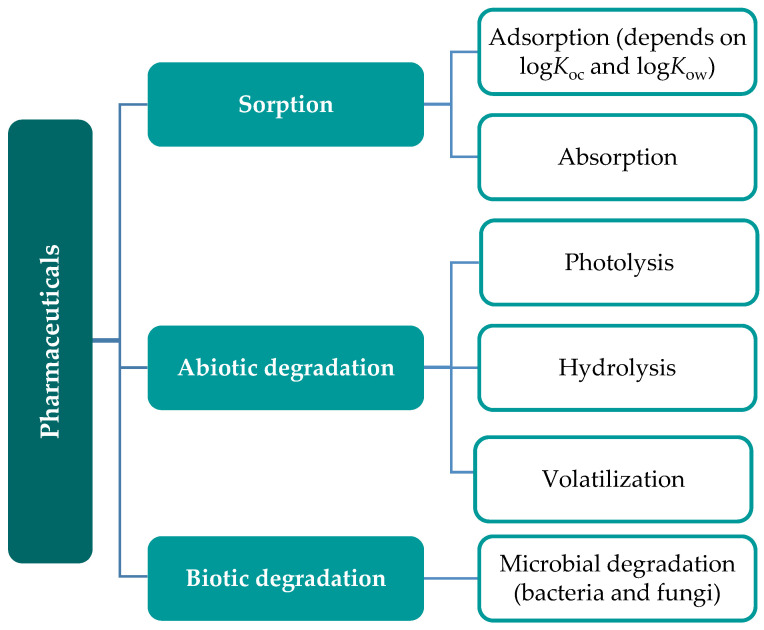
Different removal mechanisms of pharmaceuticals that can occur in the environment.

In this review, concerns regarding the occurrence and fate of pharmaceuticals, as well as their potential effects in different environments and in non-target organisms, are addressed. Among the different classes of pharmaceuticals, anti-inflammatory drugs, antidepressants, antibiotics and blood lipid lowering agents were chosen for a more accurate review, as they are among the most prescribed and consumed pharmaceuticals [22,23,24]. Autochthonous bioremediation, using native bacteria, is raised as a solution for the recovery of contaminated environments, which involves exploring and enhancing natural biological mechanisms for the removal of pharmaceuticals (Figure 1). Several case studies showing the potential of this technology for further applications are presented.

## 2. Pharmaceutical Compounds

Pharmaceuticals are natural or synthetic compounds used in human and veterinary medicine to treat or prevent several diseases. These compounds are usually polar molecules, with more than one ionizable group and different structures and functions, tending to be lipophilic or moderately soluble in water [19]. Pharmaceuticals have the ability to pass through cellular membranes and to remain as active molecules when excreted to the environment [19].

Pharmaceuticals are divided into 24 therapeutic classes, which comprise around 10, 000 different pharmaceuticals containing about 3000–4000 different active ingredients [25,26]. The most consumed ones are antibiotics, anti-inflammatories, analgesics, antidepressants, antiepileptics, lipid-lowering drugs, β-blockers, antiulcer drugs and antihistamines [22,27]. The persistence of pharmaceuticals in the environment, together with their extensive and growing use and production, continuous environmental input at low concentrations (ranging from ug L^−1^ to ng L^−1^) and potential toxicological effects on non-target organisms [20,28,29,30] have become an issue for the scientific community. In addition, the deleterious effects that these compounds may have on ecosystems’ functions and structures and on human health has also been raising concerns [1,21,31]. Experimental studies show that pharmaceuticals may cause endocrine disruption, change the structure and key functions of natural microbial communities, negatively affect invertebrates and fishes and, in the case of antibiotics, lead to the development of antibiotic resistant genes and bacteria [32,33,34,35,36,37,38]. As an example, a sublethal dose of mianserin, a tetracyclic antidepressant, promoted a significant inhibition of the growth of *Danio rerio* larvae (zebrafish), altering their physiological and biochemical parameters [39]. In another study, oxidative stress, inhibition of liver enzymes, genotoxicity and changes in steroid hormones (among others) were reported in *Rhamdia quelen* (catfish), due to paracetamol exposure [40].

The available data on the environmental presence of pharmaceuticals is unsatisfactory in terms of understanding which compounds present the highest threats to the environment. As such, it is necessary and recommended to perform more representative acute and chronic toxicity tests of pharmaceuticals in a representative range of aquatic organisms. A study reported cyto-genotoxic effects on zebra mussels due to short-term exposure to a nonsteroidal anti-inflammatory drugs (NSAID) mixture that caused an increase in oxidative stress, which in turn led to genetic damage [41]. The authors also highlighted that the observed cyto-genotoxic damages were higher when NSAID was administered as a mixture than as a single NSAID compound [41]. In another study, the Hazard Quotients (HQ) for several pharmaceuticals found in the Apatlaco River (Mexico) were observed throughout different trophic levels (*daphnia*, algae and fish) [17] and it was concluded that sensitivity to adverse effects is dependent on species and group of compounds. *Daphnia* was more sensitive to bezafibrate, acetaminophen, carbamazepine and naproxen among several other compounds [17], whereas algae displayed more negative effects to sulfamethoxazole, indomethacin and trimethoprim, while atenolol, gemfibrozil, diclofenac, ibuprofen and salicylic acid were the most toxic pharmaceuticals to fish [17]. Rivera-Jaimes and collaborators also concluded that the high concentrations of ibuprofen, sulfamethoxazole, diclofenac and naproxen found in the river water might pose a relevant risk to the whole aquatic ecosystem [17].

With the increasing evidence of the negative effects of pharmaceuticals on the environment and aquatic life, more attention has been paid to this issue. Despite the lack of regulation regarding the presence of most pharmaceuticals in aquatic environments, some of these compounds have already been included in the second and third Watch List, as is the case for (i) the antibiotics amoxicillin, ciprofloxacin, erythromycin, clarithromycin, azithromycin, sulfamethoxazole and trimethoprim; (ii) the hormones 17-Alpha-ethinylestradiol (EE2), 17-Beta-estradiol (E2) and estrone (E1); (iii) the synthetic hormone norethisterone; (iv) the antidepressant venlafaxine; and (v) three antifungal pharmaceuticals, clotrimazole, fluconazole and miconazole [42,43].

## 3. Pharmaceuticals in the Environment

The presence of pharmaceuticals in the environment has been widely reported in the past decades, being a worldwide issue of increasing concern [44]. Development and the improvement of analytical techniques has allowed the detection of pharmaceuticals at environmentally relevant concentrations with more sensitivity and precision. Gas chromatography–tandem mass spectrometry (GC–MS/MS) and liquid chromatography–tandem mass spectrometry (LC–MS/MS) are the top technologies used for the detection and monitoring of CECs in the environment, allowing the detection of CECs at ng L^−1^ and the identification of both parent compounds and associated metabolites. LC–MS/MS allows simultaneous determination of different types of compounds in highly polluted aquatic environments [45] in a unique run, within a short time period and with low costs [46]. However, the detection of transformation products or active metabolites resultant from the target pollutants is also of great relevance, as these can have a higher negative effect in the environment than the parent compound [47]. Nevertheless, the detection of these metabolites, and sometimes of the pollutant itself, can be very difficult due to the unavailability of chemical standards for all pollutants [47]. Generally, analytical techniques are combined with modern extraction and clean-up procedures [22] to assure a better analysis in terms of sensitivity and removal of matrix interferences. Solid phase extraction (SPE), liquid–liquid extraction (LLE), solid phase microextraction (SPME) and liquid–liquid membrane extraction (LLME) are the most used extraction methodologies, where the pre-concentration of the sample and the extract clean-up are obtained simultaneously from complex aquatic matrices [48,49].

Mixtures of pharmaceuticals, along with their active metabolites, are being unceasingly introduced into the environment through wastewater and sewage treatment plants, effluents from municipalities, hospitals, livestock and pharmaceutical industries, illegal untreated effluent discharges, improper disposal of unused or expired pharmaceuticals, manufacture spill accidents, manure and sludge use as organic fertilizer, treatment of crop diseases and sometimes through leachates from solid waste landfills [28,34,36,45,50,51,52]. Pharmaceuticals and their metabolites have been reported to occur in surface waters (Table S1), groundwaters (Table S2), coastal marine waters (Table S3), water for human consumption (Table S4), soils and sediments (Table S5) [20,35,53]. However, very little attention has been paid to the metabolites and transformation products resultant from the metabolism of these compounds [54]. Therefore, monitoring programs should also include the analysis of these molecules as they can be more toxic and persistent than the parent compound, and also induce negative effects on aquatic organisms and ecosystems [19,21,55,56].

One of the main concerns related to the presence of pharmaceuticals in the environment is their usual occurrence as complex mixtures rather than as a single compound, hindering the development of clean-up technologies for their removal from contaminated sites. The various pharmaceuticals that are continuously and simultaneously used and released into the environment can interact synergistically [57,58], affecting non-target organisms, since they can possess similar molecular targets [28]. In addition, the environmental concentration of pharmaceuticals, as well as their synergistic and antagonist effects, are directly related to geographical area, climatologic conditions and the occurrence of wastewater discharges [59].

Once in the environment, the persistence of pharmaceutical products can be influenced by (i) environmental factors (pH, soil characteristics, temperature, light incidence), (ii) physicochemical properties of the molecule (solubility (expressed by the octanol–water partition coefficient, (K_ow_)), molecular structure, polarity, pK_a_ or pK_b_ (in the case of having acid or base character), photo-stability, chemical stability, volatility (expressed by the Henry law constant (K_H_)), (iii) presence of other pharmaceuticals in the same matrix and (iv) presence and activity of microorganisms with the ability to degrade pharmaceuticals, metabolizing them as carbon sources or as co-metabolites [1,20,60,61,62,63]. The presence of other biodegradable organic carbon sources can improve the removal/degradation of pharmaceutical compounds by enhancing the growth of degrading microorganisms or co-metabolic processes [64,65,66].

The current concentration of pharmaceuticals released into the environment exceeds its natural capability to degrade them. The natural attenuation and detoxification of pharmaceuticals in the environment can occur through sorption, hydrolysis, photolysis, dispersion, biodegradation, dilution and, more rarely, through radioactive decomposition (Figure 1) [67,68,69,70,71,72]. Nevertheless, a review of the fate of pharmaceuticals in sewage and freshwaters suggests that hydrolysis might not have a significant role in their elimination from the environment, photodegradation and biodegradation being the processes described as more relevant in that mechanism [68]. As case studies, Lin et al. (2010) described biodegradation as a key removal mechanism for acetaminophen, an analgesic and antipyretic drug, in natural aqueous systems; however, for propranolol and acebutolol removal (both used to treat hypertension), sorption was the dominant mechanism [67]. Fluoxetine, an antidepressant, was reported to be removed from surface waters due to natural depuration by microbial communities, with an estimated half-life of 6 to 10 days [73,74]. However, this effect was not observed by Rúa-Gómez and Püttmann (2013) for the antidepressant venlafaxine, which showed slow biotic degradation in surface waters [75]. Similar results were obtained by Aymerich et al. (2016), where venlafaxine did not display natural attenuation [76]. Venlafaxine and fluoxetine displayed different behaviours in natural environments such as surface waters. The concentration and chemical structure of the two compounds, as well as the natural microbial community that was subjected to the experiment are features that influence the natural depuration of pharmaceuticals and change the behaviour of pharmaceuticals within the same family of compounds.

## 4. Therapeutic Classes of Pharmaceuticals: Presence and Effects in the Aquatic Environment

### 4.1. Antibiotics

Antibiotics are one of the most used group of pharmaceuticals in human and veterinary medicine. These pharmaceuticals are divided in several classes, such as quinolones (and fluoroquinolones), tetracyclines, macrolides, sulfonamides and β-lactam [33,77]. Fluoroquinolones, macrolides and aminoglycosides are frequently the most prescribed antibiotics in human medicine, while penicillins, tetracyclines and macrolides are the most frequently prescribed antibiotics in veterinary medicine [78]. Nearly 250 antibiotics have hitherto been registered in human and veterinary medicine [77]. The origin of antibiotics can be natural (usually products from the secondary metabolism of fungi or bacteria), semi-synthetic (by-products derived from natural products) or synthetic [20].

According to the European Centre for Disease Prevention and Control (ECDC) reports, the overall antibiotic consumption in the European Union and European Economic Area (expressed in defined daily doses (DDD) per 1000 inhabitants and per day), between 2010 and 2014, had a significant increasing trend [79], but in 2017, the consumption of antibiotics slightly decreased in some countries (Finland, Germany, Italy, Luxembourg, Netherlands, Norway, Sweden and the UK). Penicillins, quinolones, cephalosporins and other β-lactam and macrolides were the antibiotics most consumed between 2013–2017 [80]. In the 2019 report, eleven countries (Austria, Belgium, Finland, Germany, Italy, Luxembourg, Netherlands, Portugal, Slovenia, UK and Sweden) displayed a significant decreasing trend on total antibiotic consumption for systemic use, while Bulgaria, Iceland, Latvia and Ireland displayed a significant increasing trend [81].

In Portugal, amoxicillin and amoxicillin + clavulanic acid (penicillin), azithromycin (macrolide) and ciprofloxacin (fluoroquinolone) were the antibiotics most consumed between 2012–2016 [82]. Also, according to the Organization for Economic Cooperation and Development (OECD) Health Data report of 2015, Portugal registered the 12th highest volume of antibiotics prescribed between 2000 and 2013. On the other hand, Turkey, Greece, France and Italy registered the highest consumption levels [83], a tendency that has generally been maintained in the past several years [84].

Antibiotics have been detected in different environmental matrices such as soil, freshwater, seawater, groundwater and even in human consumption water, at different concentrations (Tables S1–S7). For instance, ciprofloxacin, a fluoroquinolone used worldwide, was found in surface water [85,86,87] (Table S1), groundwater [88] (Table S2), in sediments, at lower concentrations [87,89,90] (Table S5) and in wastewaters sludge [91,92] (Table S6). Erythromycin, a macrolide antibiotic, was found in surface water [87,93] (Table S1), drinking water [94] (Table S2), groundwater [88] (Table S4) and wastewater sludge [95] (Table S6).

The presence of antibiotics in the environment and their improper use has become an issue of increasing awareness and concern, since they promote bacterial resistance. This phenomenon can occur through several complex mechanisms, namely, via intracellular modification and/or deactivation of the antibiotic, exclusion of the antibiotic by the cell membrane, intracellular sequestration, reduction of cellular target sensitivity and extrusion from the cell [35]. Very low or sub-inhibitory antibiotic concentrations, comparable to those found in environmental reservoirs (water matrices and soil), can potentiate the selection of resistant bacteria and the horizontal exchange of mobile genetic elements (MGEs) encoding antibiotic resistance genes [35]. In addition, antibiotics can decrease denitrification rates, affect methanogenesis and sulphate reduction processes and induce the death of and/or inhibit degrading microorganisms in sewage treatment plants, soil and water ecosystems [20], which can have deleterious effects on the ecosystems.

### 4.2. Nonsteroidal Anti-Inflammatory Drugs

Nonsteroidal anti-inflammatory drugs (NSAIDs) are a very heterogenous group of pharmaceuticals, extensively used all over the world to treat a huge number of common acute and chronic inflammatory processes [96,97]. NSAIDs are commonly used to treat symptoms of inflammation and pain, to relieve fever and sometimes, depending on the substance, to treat rheumatic diseases [98]. NSAIDs are weak acids that act as non-selective inhibitors of one of two cyclooxygenase isoforms, COX-1 and COX-2, enzymes involved in the biosynthesis of prostaglandins that mediate pathogenic processes, including the inflammatory reaction [97,99,100,101].

Among the different classes of pharmaceuticals, NSAIDs are one of the most-used in therapeutics, not only in terms of prescriptions but also in terms of self-medication, mainly because of their low prices and over-the-counter accessibility [97,102]. The consumption of NSAIDs has been increasing 11.9% each year in UK, USA, France, Italy, Spain and Japan; this is equivalent to approximately 30 million people consuming NSAIDs every day [103]. In Portugal, more than 6 million packages of NSAIDs were consumed in 2016, ibuprofen and diclofenac being among the most-used NSAIDs [82].

The intensive consumption of NSAIDs leads to their significant detection in wastewater treatment plant effluents and consequent release to the aquatic environment due to their inefficient removal in those wastewater treatment plants [68,104]. These pharmaceutical compounds are the most frequently detected in the aquatic environment [104], representing 15% of the total drugs detected in monitoring studies worldwide. Some anti-inflammatory drugs such as ketoprofen, fenoprofen, naproxen, mefenamic acid, diclofenac and ibuprofen, were found in the aquatic environment at µg L^−1^ levels, wherein a significant portion comes from wastewater facilities [68]. The NSAIDs diclofenac, naproxen and ibuprofen have been detected in surface waters (Table S1) [87,93], seawaters [105] (Table S3), groundwaters [88] (Table S2), drinking water [94,106] (Table S4), wastewater, sludge [93,107,108] (Tables S6 and S7) and sediments [87,109] (Table S5).

Several studies reported the effect of NSAIDs in non-target organisms [110,111]. In a study conducted by Xia et al. (2017), a significant hatch delay in zebra fish (*Danio rerio*) due to the suppression of overall embryo motion was observed after an exposure to ibuprofen (500 µg L^−1^) and diclofenac (5 µg L^−1^ and 500 µg L^−1^) [110]. Kwak et al. (2018) described a reduction in the reproduction of the crustaceans *Daphnia magna* and *Moina macrocopa* due to chronic exposure to naproxen [111]. In addition, the same authors reported a decrease in the survival of juvenile *Oryzias latipes* fish exposed to 5 mg L^−1^ of naproxen [111]. Ibuprofen was found to cause nephrotoxicity in the south American catfish, *Rhamdia quelen* [112]. When exposed to four different NSAIDs, ibuprofen (racemic and S-(+)- ibuprofen), ketoprofen and aspirin, the green algae *Scenedesmus obliquus* showed growth inhibition, severe damage to cellular structures and significant effects on photosynthesis and on the PSI–PSII photosynthetic electron transport chain as well as on carbon assimilation and photorespiration [113]. Authors also reported that ketoprofen was the NSAID that exerted higher toxicity on *Scenedesmus obliquus*, suggesting that it could be related to its high liposolubility and bioavailability (Wang et al., 2020).

### 4.3. Antidepressants

Antidepressants are an important group of pharmaceuticals designed to treat psychological disorders, and extensively used throughout the world. Antidepressants can be divided into four major classes: monoamine oxidase inhibitors (MAOIs), tricyclic antidepressants (TCAs), norepinephrine reuptake inhibitors (SNRIs) and selective serotonin reuptake inhibitors (SSRIs) [114,115]. From these, SSRIs are the most-prescribed class of antidepressants [114,116]. SSRIs have been used since the 1980s [117] and continue to be the first choice in treating depression due to their therapeutic effectiveness and higher acceptability and safety compared with other groups of antidepressants (for example, TCAs or SNRIs) [118]. Antidepressants are used to treat clinical depression, obsessive-compulsive disorder, panic disorder, attention-deficit disorder, eating disorders (nervous bulimia and compulsive ingestion) and social phobia [117,119]. The SSRIs fluoxetine, paroxetine, citalopram and sertraline and the SNRIs venlafaxine and duloxetine are examples of the currently most-prescribed antidepressants [116,120,121]. SSRIs, SNRIs and TCAs act through modulation of serotonergic, dopaminergic or noradrenergic neurotransmission [121].

Consumption of antidepressants has been increasing in the past few years. In fact, reports from OECD show that between 2000 and 2017, the consumption of antidepressants doubled in OECD Countries [84]. Moreover, data from 2017 showed that Iceland and Canada presented the highest consumption of antidepressants, while Latavia, Korea, Hungary and Estonia presented the lowest consumption values [84]. In Portugal, around 300,000 packages of antidepressants were prescribed in 2001, and in 2016, almost 8,000,000 packages of antidepressants were consumed (data does not include the antidepressants prescribed in hospital facilities) [82]. According to the 2019 OECD report, Portugal was the 4th country with the highest level of antidepressant consumption between 2000 and 2017, only surpassed by Iceland, Australia, Canada and UK [84], a tendency also reported in the 2015 OECD report [83]. In the past few years, the continuous growth of antidepressants consumption was linked to economic crises. Specifically, in Portugal, antidepressant consumption went up by 30% between 2007 and 2012, but this level was lower than the 60% growth rate observed between 2002 and 2007 [122]. In Spain, the consumption of antidepressants per capita increased by 23% between 2007 and 2012, even though this increase was lower than the 44% growth rate observed between 2002 and 2007 [122]. During this period, both countries faced financial and economic adversities, expressed by an increase in unemployment (3% for Portugal and 12% for Spain), the fear of losing employment and significant reductions to or freezing of salaries, among others [123]. According to Karanikolos et al. (2013), the economic crisis had a significant impact on mental health, translating into an increase in antidepressant consumption [123]. Nevertheless, in Germany, one of the countries less affected by economic crises, a quick rise in antidepressants consumption (over 12%) was observed between 2007 and 2012 [122]. Data on antidepressant consumption may be, however, underestimated (based on prescribing trends), as some antidepressants, like fluoxetine, are off-patent, thus being more difficult to track [124]. Nowadays, with the SARS-CoV-2 (COVID-19) pandemic situation, a new increase in the consumption of these pharmaceutical products can be expected. The COVID-19 pandemic is a challenge to both the physical and mental health of the human race, as well as to the economy and social life [125]. The implementation of lockdown measures, including work disruptions, school closures and physical distancing, might increase social isolation and loneliness, both in turn associated with increased anxiety, depression and suicidal behaviour [125].

Antidepressants can enter aquatic ecosystems through inefficient treatment of wastewaters from municipalities, hospitals and pharmaceutical industries and through improper disposal. The presence of antidepressants in different environments (wastewaters, surface waters and/or drinking water) was reported in several studies [73,105,108]. Antidepressants such as fluoxetine, paroxetine, sertraline, venlafaxine and citalopram have been detected in surface waters [87,105] (Table S1), wastewaters [86,105,108] (Table S7), sludge [108] (Table S6) and sediments [87] (Table S5). Some of them were also detected in seawater [105] (Table S3) and groundwater [88] (Table S2). For instance, venlafaxine was detected in surface waters at concentrations ranging between 1.15 and 575 ng L^−1^ [85,87] and at lower concentrations in seawater (52 ng L^−1^) [105], sediments (0.05–1.94 ng g^−1^) [87] and sludge (37.9 ng g^−1^) [108]. The antidepressant paroxetine was reported in surface waters at concentrations ranging between 0.27 and 40 ng L^−1^ [87,105] and in groundwater at similar concentrations (5.17–30.2 ng L^−1^) [88]. Similarly to other pharmaceuticals, a range of concentrations of antidepressants can be found in the environment (Tables S1–S7). This variation can be related to the consumption profile associated with the site where the samples are being collected, detection methods used for their detection and, more importantly, with the behaviour of each antidepressant in the environment.

Antidepressants can induce effects in living organisms even at very low concentrations, so their presence in the environment is of high concern [124,126]. This problem can be exacerbated through the chronic administration of antidepressants, which may lead to a higher and continuous environmental input and exposure to these compounds [127]. For example, serotonin is known to regulate several physiological processes in fish, mollusks and protozoans [127]. Several studies have addressed the adverse effects of antidepressants in organisms. Johnson et al. (2007) showed that the SSRIs fluoxetine, fluvoxamine and sertraline presented toxic effects on algae, with IC10 values ranging from 4.6 to 6100 μg L^−1^ (depending on the algae species) after 96 h acute growth inhibition [128]. Sehonova et al. (2019) studied the effects of three antidepressants, venlafaxine, amitriptyline and sertraline, on early life stages of non-target aquatic organisms (*Danio rerio* and *Xenopus tropicalis*), showing swimming alterations at high antidepressants concentration (i.e., concentrations higher than those found in the environment) [115]. In addition, lethal and sublethal effects were observed in the embryos of both species for the highest tested amitriptyline concentration. The study also reported that, at environmentally relevant concentrations, the three antidepressants were suspected to have an effect on mRNA expression of genes related to heart, eye, brain and bone development [115]. Nowakowska et al. (2020) showed that exposure of zebra fish (*Danio rerio*) larvae to selected antidepressants (paroxetine, sertraline, fluoxetine and mianserin) caused an increasing rate of abnormal embryo and larvae development, accelerating the hatching time and influencing the total hatching rate [129]. The authors also reported a decrease in the proliferation of hepatocytes in larvae previously subjected to paroxetine, mianserin, sertraline (10 μg L^−1^) and also to a mixture of all the antidepressants at 25 μg L^−1^ [129].

### 4.4. Blood Lipid Lowering Agents

Blood lipid lowering agents are commonly prescribed to treat diseases related to cardiovascular disorders [130]. There are two main groups with different functions: statins and fibrates. Statins are used mainly to suppress cholesterol biosynthesis by inhibiting the 3-hydroxy-3-methylglutaryl coenzyme A (HMG-CoA) reductase [131]. Several statins are currently available on the market, such as atorvastatin, simvastatin, lovastatin, pitavastatin, fluvastatin, cerivastatin, rosuvastatin and pravastatin [132,133]. On the contrary, fibrates are used to reduce plasma levels of fatty acids and triacylglycerol by stimulating β-oxidation of fatty acids, mostly in peroxisomes and partly in mitochondria [131]. Examples of some well-known fibrate drugs are fenofibrate, bezafibrate, ciprofibrate and gemfibrozil [131].

Lipid lowering agents are widely prescribed worldwide and their consumption has been growing. Prescriptions of cholesterol-lowering drugs have grown between 2000 and 2013, with the Slovak Republic, UK and Australia being the countries with the highest consumption per capita in 2013 [83]. The same tendency was reported in the latest OECD report, in which the UK, Denmark and Belgium had the highest consumption per capita in 2017 [84]. In the case of Portugal, more than 10 million packages were consumed in 2016, the statins simvastatin, atorvastatin and rosuvastatin being the most-consumed ones [82]. Fenofibrate and bezafibrate were also widely consumed in Portugal in the same year but in smaller proportions compared to statins [82].

Like other pharmaceuticals, lipid-lowering agents have been detected in different environmental compartments. As an example, bezafibrate was detected in effluent samples from three different wastewater facilities in Spain, at concentrations ranging between 2 and 132 ng L^−1^ (Table S7), and in three estuarine environments (water samples) at concentrations ranging between 2 and 67 ng L^−1^ [134] (Table S1). Surface waters in Portugal were found to be contaminated with low levels of bezafibrate and gemfibrozil (11.86–15.52 ng L^−1^ and 6.69–10.34 ng L^−1^, respectively) [135]. In Mexico, bezafibrate and gemfibrozil were detected in surface water at levels ranging between 265–2100 ng L^−1^ and 9–380 ng L^−1^, respectively [17]. Fibrates have also been detected in groundwater [88] (Table S2) and seawater [105] (Table S3). Regarding the environmental presence of statins, few studies have been dedicated to this issue, despite the increase in their consumption. In a study conducted by Langford and Thomas (2011), simvastatin was not detected in surface water nor in sediments (concentrations below the detection limit); nevertheless its metabolite, simvastatin hydroxy carboxylic acid, was present in surface waters (27–66 ng L^−1^ ) and in sediments (2–4 ng g^−1^) [136]. In the same study, the metabolites of atorvastatin, p-hydroxy atorvastatin and o-hydroxy atorvastatin, were detected in wastewaters at levels ranging between 83–233 ng L^−1^ and 60–169 ng L^−1^, respectively, whereas the parent compound was detected at lower levels (23–37 ng L^−1^). Atorvastatin and rosuvastatin were detected in 11 wastewater treatment plants in Ontario, in both influent and effluent samples [137]. Atorvastatin was detected at concentrations ranging between 72–263 ng L^−1^ and 10–122 ng L^−1^ in influent and effluent samples, respectively, and rosuvastatin was detected at 34–604 ng L^−1^ and 190–552 ng L^−1^ in influent and effluent samples, respectively (Table S7) [137].

The effects of blood lipid lowering agents on the environment and living organisms have been reported in some studies. Mijangos et al. (2018) evaluated the environmental risk of different pharmaceuticals in wastewater effluents and estuarine samples, by analyzing the chronic and acute toxicity of selected pharmaceuticals [134]. In terms of chronic toxicity, the authors showed that: (a) bezafibrate, alongside diclofenac and sulfadiazine, and (b) bezafibrate and diclofenac displayed the most negative impact on wastewater effluents and estuarine sediments, respectively (risk quotient RQ > 1) [134]. However, in terms of acute toxicity, bezafibrate presented a RQ <1 for both matrices [134]. In a study with mussels (*Mytilus edulis*), atorvastatin induced an increase of the basal metabolic rate and a reduction of energy reserves [138]. In addition, the authors also reported that atorvastatin can act as a metabolic disruptor and chemosensitizer in *M. edulis*. Barros et al. (2018) showed that simvastatin exposure led to a reduction in cholesterol/triglyceride levels and altered key gene expression in zebra fish (*Danio rerio*) [139].

## 5. Removal of Pharmaceuticals in Wastewater Treatment Plants and Factors That Can Affect Their Removal

Wastewater treatment plants (WWTPs) were designed to efficiently remove suspended solids, organic matter, nutrients and pathogens [22]. However, their efficiency in remove micropollutants, such as pharmaceuticals, is generally very low, as they were not designed to remove these types of compounds [20,22]. Pharmaceuticals can go through conventional wastewater treatments unaltered because of their moderate to high solubility and their resilience to degradation during biological and chemical processes [103,140].

The inefficient removal of pharmaceuticals in WWTPs can be related to several factors inherent to treatments and operational conditions. One major factor that cannot be controlled is weather conditions. The removal efficiencies can be lower during winter due to heavy rainfall and lower water temperature, which can lead to a decrease in biodegradation kinetics. Moreover, pollutant concentration can affect the removal rates. For instance, the removal rates of some anti-inflammatories, antibiotics and antidepressants can decrease in winter, since usually, at this time of the year, the consumption of these compounds increases due to weather-associated health problems, such as flue, rheumatic pain [141,142] or seasonal affective disorder (conditions that have more incidence during specific times of the year, usually in autumn and winter) [143,144]. Vieno et al. (2005) reported that the total concentration of the pharmaceuticals ibuprofen, naproxen, ketoprofen, diclofenac and bezafibrate in the effluents of a sewage water treatment plant was 3–5 times higher in winter (about 2500 ng L^−1^) than in the other seasons (about 500−900 ng L^−1^) [145]. On the other hand, Guerra and co-authors studied six WWTPs and found seasonal differences in terms of pharmaceutical concentrations in the effluents of only one WWTP [22].

Other factors such as pH, hydraulic retention time (HRT), sludge retention time (SRT), food to microorganisms (F/M) ratio and the configuration of WWTPs, can have a key role in the removal efficiency of pharmaceuticals [68,146,147]. HRT and SRT control reaction time and loading, affecting biomass activity and the adaptation of different microbial communities [146,148]. It is expected that higher HRT and SRT lead to higher biodegradation of contaminants, because higher retention times can promote the development of slowly growing bacteria and, thus, stimulate microbial diversity with wider physiological capabilities [149]. Indeed, Clara et al. (2005) showed that the removal of different emerging pollutants, including the pharmaceuticals bezafibrate and ibuprofen, was enhanced at higher SRT but, for other compounds such as carbamazepine, the same effect was not observed, as carbamazepine did not degrade during the treatment [149]. Guerra and co-authors reported that the efficient removal of several pharmaceuticals and personal care products was strongly related with summer temperatures, HRT longer than 16 h and nitrifying activity [22]. Fernandez-Fontaina et al. (2012) studied the influence of HRT, SRT, temperature and nitrifying activity on the biodegradation of several contaminants, including different classes of pharmaceuticals, in a pure nitrifying reactor [150]. The authors observed that the biodegradation rates of ibuprofen, naproxen, trimethoprim, roxithromycin, erythromycin and fluoxetine (and other emerging pollutants) increased with an increase in nitrogen loading rates, the ammonium monooxygenase enzyme (AMO) being responsible for co-metabolic biodegradation [150]. They also reported that contaminants with slow or intermediate degradation kinetics, like fluoxetine or antibiotics, are expected to have lower biodegradation efficiencies when HRTs are lower and/or loading rates are higher [150]. Despite these results, other authors reported that there was no clear relationship between removal efficiencies and SRT/HRT for the antibiotics ciprofloxacin, ofloxacin and norfloxacin, beta-blockers [151] and carbamazepine [151,152].

pH is another parameter that can highly influence the removal of pharmaceuticals, as under different pH conditions these compounds can change their ionic form, becoming neutral, cationic, anionic or zwitterionic. Thus, the physical–chemical and biological properties of pharmaceuticals, such as toxicity, activity, sorption and photosensitivity, will vary with the pH of the medium [146]. Antibiotics are one of the groups that can be strongly affected by pH variations, especially the antibiotics ciprofloxacin, tetracycline and penicillin G [146]. Tadkaew et al. (2010) studied the removal of several pharmaceuticals in a submerged membrane bioreactor (MBR) at different pH values (between 5 and 9) and showed a strong influence of pH in the removal of ibuprofen, ketoprofen, diclofenac and sulfamethoxazole, with the highest removals being achieved at pH 5 [153]. On the other hand, in a different study, but for the same pH range, no significant influence of pH on the removal of the anti-lipidic bezafibrate, in an activated sludge system, was observed [154]. Baena-Nogueras et al. (2017) showed that pH has a key role in the photodegradation of many pharmaceutical compounds [155]. In fact, the authors observed that photodegradation of the analgesic acetaminophen was higher at pH 4 or 9 compared with neutral pH (pH 7) but for other pharmaceuticals like diclofenac, ketoprofen and ibuprofen (NSAIDs pharmaceuticals), no significant changes were observed [155].

The F/M ratio can affect organic removal efficiency, microbial composition and sludge properties [156,157]. Lower F/M ratios combined with higher retention times can lead to an increase in biodiversity and enhance the degradation of pollutants by co-metabolism [158,159]. In addition, low substrate availability can induce microorganisms to use poorly degradable compounds as carbon sources [146].

Treatment configuration also has a huge impact in the removal efficiencies of pharmaceuticals in WWTPs [147]. Different biochemical environments (aerobic, anaerobic or anoxic conditions) can promote or inhibit the removal of certain pharmaceuticals. The microbial communities present in each type of environment are completely different and have different metabolic mechanisms, which can influence the biodegradation of the pollutant. Alvarino and co-authors (2014) studied the fate of 16 pharmaceutical and personal care products (PPCPs) in an aerobic conventional activated sludge reactor (CAS unit) and in an upflow anaerobic sludge blanket reactor (UASB reactor), testing different operational periods [160]. The authors showed that under aerobic conditions (CAS unit), higher removal efficiencies were obtained for most of the 16 PPCPs [160], except for sulfamethoxazole and trimethoprim. However, in the CAS unit, carbamazepine, diclofenac, diazepam and trimethoprim had removal efficiencies below 10% in all tested periods [160]. In addition, the authors also reported that, under anaerobic conditions (UASB reactor), sulfamethoxazole and trimethoprim presented higher removal efficiency, while the removal efficiency of naproxen was similar in both reactors [160]. Suarez et al. (2010) reported improvements in ibuprofen and diclofenac removal from wastewater effluents only when specific types of bacteria were able to grow [161].

The fate of pharmaceuticals during wastewater treatment processes can also differ with therapeutic class. In a study that evaluated the fate of several pharmaceuticals in WWTPs, it was reported that anti-inflammatories and analgesics were susceptible to biodegradation in a conventional biological treatment, whereas they were not biodegraded during a chemically assisted primary treatment [22]. The study also showed that antibiotics and antifungal compounds were highly resistant to both treatments, having been detected at high concentrations in the treated effluent and sludge [22].

Despite all these factors, the removal efficiencies of pharmaceuticals in WWTPs can be different in different facilities using the same treatments or even in the same facility and on the same day. These changes can occur due to physicochemical properties of pharmaceuticals, effluent composition (microbial community, wastewater composition and other elements that can be present and improve or inhibit degradation) and different WWTPs configurations like biological treatment configuration and operational parameters. For instance, the same pollutant can have different removal rates in the same type of biological treatment and even in the same facility. A study conducted by Roberts and co-authors showed that for some target pharmaceuticals, removal efficiency varied between sampling campaigns at relatively constant sewage treatment conditions [162].

All the mentioned factors can be optimized, and more attention is needed regarding this topic. An improvement in these parameters can lead to better removal efficiencies of some pharmaceuticals and other organic compounds in wastewater treatment facilities without changing the type of treatment, leading to lower emissions of pharmaceuticals in the environment.

Several technologies have emerged in the past few years to tackle pharmaceutical contamination and improve their removal in WWTPs, since these are the main inputs of pharmaceuticals into the environment. By solving the problem in wastewater facilities, a large amount of pharmaceuticals can be removed before they enter the environment. Chemical-based technologies have been developed, in which advanced oxidation processes are the most-studied [49]. Chlorination, ozonation, UV treatment, electrochemical oxidation and Fenton and photo Fenton oxidation are examples of advanced oxidation processes [49,163]. Physical-based technologies have also been explored, with adsorption processes (activated carbon, carbon nanotubes) being the most commonly known [49]. A sustainable alternative to be considered for the removal of pharmaceuticals from wastewaters are constructed wetlands (CWs). This technology, designed to mimic natural wetland habitats and their important interactions, can be used as secondary or tertiary treatment [164,165]. The potential of CWs for the removal of pharmaceuticals has been reported by different authors [37,164,166].

## 6. Bioremediation Processes as a New Remediation Technology

The degradation of pollutants by microbial communities is one of the most important mechanisms for removing these compounds from the environment. Microbial communities are essential degraders of organic matter and, at the same time, they provide nutrients to other organisms in the food chain [20]. Therefore, they are extremely important for the function, maintenance, quality state and natural depuration of ecosystems. When a xenobiotic enters the environment, changes in local microbial communities can occur and, consequently, ecosystem processes can also change [69]. Microbial communities can degrade organic contaminants by metabolic and co-metabolic reactions, with the latter being the most important for the elimination of pollutants [20,69]. Bioremediation technology relies on the metabolic capacity of microorganisms to degrade pollutants, taking advantage of natural detoxification processes [167]. For this, the selection and isolation of natural degrading microorganisms to develop microbial inocula able to degrade target contaminants, with minimal long-term influence in the microbial community of the contaminated sites [168] are crucial. This technology has gained attention due to the high abundance and diversity of microorganisms in nature and their highly diverse catalytic mechanisms and capacity to function in, and adapt to a multitude of conditions [167].

A consortium of microorganisms is usually more effective for the degradation of contaminants than single bacterial strains, as cooperative interactions or synergistic effects among different bacteria play a crucial role in the degradation of these compounds [49,169]. Nevertheless, bioremediation of a contaminated site is only possible if the toxicity of the contaminant does not inhibit microbial activity [20].

Bioremediation has been reported as an efficient method for the remediation of organic pollutants such as hydrocarbons [170,171,172], pesticides [66,173,174], polychlorinated biphenyls [175] and pharmaceuticals [176,177]. This technology is based on three strategies: biostimulation, bioaugmentation or a combination of both. Biostimulation aims to stimulate a local and/or introduced microbial community by adding an inorganic nutrient cocktail (mainly containing nitrogen and phosphorus) to the contaminated site, in order to avoid metabolic restrictions [178,179,180]. Bioaugmentation comprises microbial inoculation of degrading microorganisms to the contaminated site in order to enhance the biodegradation of the target pollutant(s) [178,179,180]. A combination of both processes, bioaugmentation and biostimulation, has been explored, since the addition of a nutrient cocktail for stimulation of the natural and bioaugmented community is a crucial feature for the success of the bioremediation process.

### 6.1. Biodegradation of Pharmaceuticals by Single Strains

Biodegradation of pharmaceuticals using single bacterial strains isolated from different environments has been investigated. Ofloxacin, norfloxacin and ciprofloxacin were reported to be biodegraded, individually or as mixture, in the presence of sodium acetate as an additional carbon source [181], by a bacterial strain identified as *Labrys portucalensis* F11 [182], isolated from an industrially contaminated site in Northern Portugal. Complete degradation by *L. portucalensis* F11 of 2 µM racemic fluoxetine (FLX) in 30 days was reported by Moreira et al. (2014), however, when the racemic compound was supplied at 4 µM, the (R)-enantiomer was preferentially degraded over the (S)-enantiomer, with 80% of (S)-FLX and 97% of (R)-FLX being degraded [183]. These findings indicated that enantiomeric pharmaceuticals are not biodegraded at the same extent. The authors also reported a decrease in the removal rate of FLX with the increase in its concentration, a result also reported by Amorim et al. (2014) [181]. Complete dehalogenation of moxifloxacin (up to 7.5 μM) by *L. portucalensis* F11 strain, with sodium acetate as co-substrate, was reported by Carvalho et al. (2016) [64]. This microorganism was also shown to be capable of fully removing 34 µM of diclofenac from the culture medium in co-metabolism with sodium acetate (5.9 mM) in 25 days [65]. In addition, no chlorinated compound was found at the end of the experiment, indicating complete dehalogenation of diclofenac [65]. The degradation of diclofenac and carbamazepine (at 10 mg L^−1^) by bacterial strains isolated from activated sludge from a municipal WWTP was also tested by Bessa and co-authors [184]. The authors reported a *Brevibacterium* sp. D4 strain capable of removing 90% of diclofenac and *Starkeya* sp. C11 and *Rhizobium* sp. C12 strains capable of removing 32% of carbamazepine, both in the presence of acetate as a supplementary carbon source [184].

The biodegradation of sulfamethazine was investigated by Pan et al. (2017), with the bacterial strains *Geobacillus* sp. strain S-07 and *Geobacillus thermoleovorans* [185]. In 24 h of the experiment, strain S-07 were revealed to be capable of removing more than 95% of the antibiotic in co-metabolism with glucose, while the type strain *G. thermoleovorans* only removed 30% of the compound (a percentage that also includes abiotic degradation) [185]. In another study, the authors investigated the degradation of ciprofloxacin by the thermophilic bacterium *Thermus* sp. strain C419, isolated from the sludge of an antibiotic-producing factory [186]. The authors tested different temperatures, ranging from 65 °C to 80 °C, and found that ciprofloxacin was better-degraded at 70 °C. In addition, the authors performed biodegradation experiments in co-metabolism with sodium acetate and observed that acetate promoted bacterial growth and enhanced the degradation of ciprofloxacin, having removed around 60% of the antibiotic after 5 days of exposure [186]. Mulla et al. (2018) assessed the potential of three bacterial isolates, *Ochrobactrum* sp. SA1, *Labrys* sp. SC11 and *Gordonia* sp. SCD14, for degrading sulfamethoxazole [187]. The three isolates were obtained from a culture enriched from wastewater and sludge inoculum and with 6 mg L^−1^ of the target compound as sole carbon source and were able to partially degrade 5 mg L^−1^ of sulfamethoxazole (45.2%, 62.2% and 51.4%, respectively) [187]. The ability to degrade paracetamol was studied using three bacterial strains, as a consortium or as single strains, one *Stenotrophomonas* sp. and two *Pseudomonas* sp., isolated from a paracetamol-degrading microbial aggregate growing in a lab-scale airlift sequencing batch reactor [188]. The three strains were able to individually degrade the pharmaceutical, however, high concentrations of the pharmaceutical were found to be toxic and to inhibit the degradation process, i.e., degradation by *Stenotrophomonas* sp. was inhibited at 600 mg L^−1^ whereas degradation by the strain *Pseudomonas* sp. f2 was inhibited in the presence of 3.000 mg L^−1^ of paracetamol [188]. However, *Pseudomonas* sp. fg-2 was able to degrade up to 2000 mg L^−1^ in 45 h [188]. In addition, they also showed that the consortium formed by the three strains was more efficient than the strains individually, since this mix was able to completely degrade paracetamol at concentrations up to 4000 mg L^−1^, indicating possible synergistic interactions between the three isolates in the degradation of the pharmaceutical [188].

### 6.2. Biodegradation by Bacterial Consortia

Studies exploring the potential of microbial consortia to degrade pharmaceuticals have also been performed. An enriched consortium from activated sludge was developed, able to use triclosan as the sole carbon source [189]. However, the strains recovered from the enriched consortium (composed by the genera *Pseudomonas*, *Alcaligenes*, *Rhodanobacter*, *Agrobacterium* and *Sphingomonas*, all belonging to the Proteobacteria phylum) were not able to use triclosan as a sole carbon source in liquid medium, either individually or combined. In another study, the role of different types of bacteria (ammonia-oxidizing and heterotrophic bacteria) in the degradation of trimethoprim and 17α-ethinylestradiol was evaluated [190]. A mixed culture of both ammonia oxidizing and heterotrophic bacteria (composition not disclosed) was proved to enhance the removal of 17α-ethinylestradiol [190]. Alexandrino et al. (2017) investigated the biodegradation of three enrofloxacin and ceftiofur concentrations (1, 2 and 3 mg L^−1^), either alone or in mixture, using microbial consortia obtained from rhizosediment of plants from constructed wetlands [177]. In that study, the authors reported complete removal of ceftiofur in all experiments, even in the presence of enrofloxacin. However, enrofloxacin never reached complete removal (around 40–55%), with the increasing antibiotic concentration being a limiting factor [177], as also reported by other authors [181,183]. The authors additionally found that the predominant microorganisms resulting from acclimation with the target antibiotics belonged to the phyla Proteobacteria (for example, *Achromobacter*, *Variovorax* and *Stenotrophomonas* genera) and Bacteroidetes (for example, *Dysgonomonas*, *Flavobacterium* and *Chryseobacterium* genera) [177]. Facey et al. (2018) showed that diclofenac was removed in seven days by two microbial consortia native to forest soils in Germany (microbial composition not identified), when present at concentrations up to 0.1 g L^−1^ [191]. Topp et al. (2008) reported the biodegradation of naproxen by microbial communities (composition not revealed) of three types of agricultural soil (sandy loam, loam and silt loam) and showed that this compound was rapidly biodegraded and mineralized [192]. More recently, studies conducted by Duarte et al. (2019) and Fernandes et al. (2020) showed the capability of five bacterial consortia, enriched with sludge or estuarine sediment, to degrade 1 mgL^−1^ of paroxetine and bezafibrate, under different incubation conditions (static and stirred) [176,193]. In this study, bacteria affiliated with the phylum Proteobacteria were dominant in all consortia, with the genus *Pseudomonas* being the most abundant [176]. Nonetheless, bacteria belonging to the genera *Acinetobacter* (Proteobacteria), *Dyadobacter* (Bacteroidetes) and *Microbacterium* (Actinobacteria), among others, were also found [176].

All these studies clearly show that the use of bacterial strains or bacterial consortia can be an option for removing/degrading pharmaceutical compounds from the environment. Despite the important information that these studies provide, it is very difficult to describe a common pattern in terms of degradation of these compounds, since wide variations in removal efficiencies across/between therapeutic classes, treatment processes and even between different studies using the same pharmaceutical compound were observed.

### 6.3. Factors Affecting Biodegradation Process

In a biological treatment, several processes, such as volatilization, adsorption and biodegradation can occur [49]. Biodegradation/biotransformation together with adsorption are the processes that have a greater role in the degradation of pharmaceuticals during biological treatment in wastewater treatment facilities [194]. The physicochemical properties of pharmaceutical compounds determine if they will be either degraded or adsorbed by the sludge. Biodegradation of these compounds is also dependent on the abundance of microbial degraders in the treatment system and can be very low in systems poor in microbial degraders [49]. In addition, pharmaceutical degradation can be affected by interactions with other compounds (antagonistic effect) [62] or interactions among microorganisms (synergistic effect) [169]. These effects can improve or inhibit the degradation of the pharmaceuticals, this being a potential explanation for the different removal efficiencies obtained with the same treatment.

To evaluate and compare the biodegradability of pharmaceuticals, it is necessary to take into account the intrinsic differences in the chemical structures of each compound, like the presence of sugar moieties or of halogens on the compound structure, which can render the compound more or less biodegradable [195]. Thus, pharmaceuticals within the same therapeutic class but with different chemical structures can have different biodegradation rates, since biodegradation processes engage enzymatic reactions that are chemically specific [1]. In addition to the factors presented here, there are others that can also affect the biodegradation of pharmaceuticals and explain the discrepancies in the removal rates observed for the same compound. The first, and a very important one, is the pharmaceutical concentration. Different concentrations lead to different removal efficiencies that cannot be compared. Moreover, too-high pharmaceutical concentrations can inhibit the microbial community and exert a toxic effect on microorganisms [1]. Another factor, which is related with the first one, is the concentration of the primary substrate. Pharmaceuticals can potentially be used as a primary substrate, i.e., they can be utilized by microbial communities as a carbon and energy source, depending on the concentration of the pharmaceutical but, if the concentration is very low, it may not be sufficient to induce specific degrading enzymes and so the compound may be preferentially biodegraded through co-metabolism [2]. So, the fact that pharmaceuticals can be used as a primary substrate or a co-substrate can contribute to differences in biodegradation rates. The third factor that can affect biodegradation rates and accentuate discrepancies in the removal of pharmaceuticals is the incubation time, which is usually arbitrary. The same compound can present different removal rates for different incubation times. Lastly, biodegradation rates can depend on source, concentration and pre-adaptation of the microbial inoculum. The removal efficiencies and lag times can be affected by these factors according to the response of the microbial community to those variables [1]. For instance, if the microbial community present in the inoculum has been previously exposed to pharmaceuticals, this community may more easily recognize the compound, allowing faster adaption of the community to the new conditions.

In summary, several intrinsic and extrinsic factors can affect biodegradation and biotransformation processes, both in natural environments and in engineered systems. More studies must be conducted to better understand and minimize the constraints that may arise in the development of bioremediation technologies. Despite the constraints that, as in any other technologies, can appear, the low negative impacts of implementation, no need for additional constructions for implementation, high efficiency and long-term viability [196] make bioremediation technology a sustainable solution that should be considered for the recovery of sites contaminated with pharmaceuticals and other pollutants.

## 7. Conclusions and Future Perspectives

**Figure 2:**
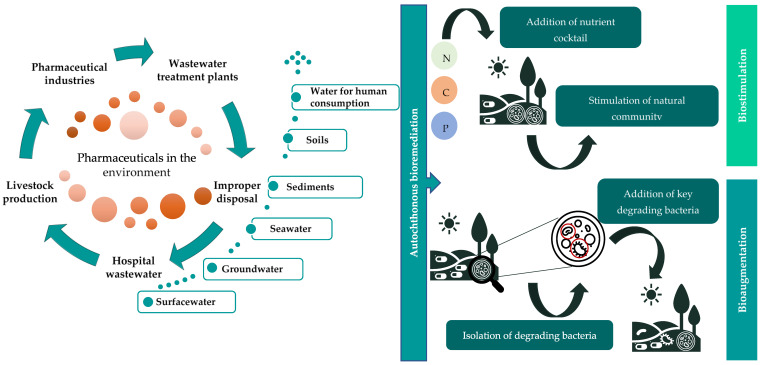
Summary regarding the input and environmental fate of pharmaceuticals in the environment and bioremediation process as a solution. C—carbon; N—nitrogen; P—phosphorous.

Pharmaceutical compounds have a prevalent role in our society and their consumption tend to increase, since they are essential for treating human and veterinary illnesses and providing a better quality of life. With their continuous consumption and manufacturing, the incessant release of pharmaceuticals into different environments is inevitable, as shown in Figure 2. After the improvement of detection methods, the next step to take is to find sustainable and efficient technologies to tackle this problem, both to prevent the environmental input of these compounds and to remove them from/recover impacted environments. There are several efficient technologies for remove pharmaceuticals, however, they are not suitable for application in natural environments. Bioremediation technologies based on microbial communities with the capacity to degrade pharmaceuticals have been presented as a possible solution, as shown in Figure 2, in which different strategies can be selected: biostimulation, bioaugmentation and a combination of both. Degradation of pollutants by microorganisms is known to be an important detoxification process in nature and it has been proved that sustainable technologies based on degrading microorganisms are a suitable solution to be developed and applied for the recovery of contaminated environments. To our best knowledge, bioremediation technology has not been applied for the removal of pharmaceuticals in natural environments, despite the increasing number of studies looking into the potential of microorganisms to metabolize/degrade pharmaceuticals. These compounds are distributed in different natural environments, such as oceans and rivers, that can represent a tremendous area to be treated. To overcome the challenge of selecting the total area from a river or other natural matrices, the technology could be designed targeting specific contaminated areas in which the pollution with pharmaceuticals is higher.

Several studies performed involving bacterial communities or single strains able to degrade different pharmaceutical classes have proved these are options for addressing pharmaceutical contamination. However, more studies are needed regarding the development and application of bioremediation technology in different environments contaminated with these compounds. There are several topics regarding the development of this technology that should be addressed in future studies. For instance, tests in natural media should be performed to investigate the effects of the addition of bacterial formula in natural communities and to evaluate if the added microbial community continues to have a high removal efficiency. In natural media, there are several factors that are continually changing and that can be a step back in the development of this technology. For example, temperature, hydraulic conditions as other physical–chemical properties can influence the communities in the contaminated site and affect the performance of the designed technology. The concomitant presence of other pollutants (metals, nanoparticles, pesticides and other pharmaceuticals with different functions and structures) should also be tested, since the presence of other pollutants can inhibit the added bacteria or exert unexpected effects that can influence the performance of this technology. Other major aspect that should be addressed is the production of metabolites generated during the degradation process. Most of the metabolites that are formed are unknown, justifying more studies on the metabolic pathways and final degradation products. The goal of the biodegradation process is that the generated metabolites become less toxic or become completely inactive. As in the case of halogenated pollutants, microbial dehalogenation is a crucial reaction, since halogen atoms are usually responsible for the environmental recalcitrance of a molecule, also increasing the chances of generating less toxic metabolites [197].

Most of the existing technologies are directed towards WWTPs, as they represent one of the main sources of pharmaceuticals in the environment. Application of technologies such as advanced oxidation processes and reverse osmosis, among others, can be considered as a tertiary treatment, in order to avoid the release of pharmaceuticals into the environment. Still, WWTPs are not the only input of pharmaceuticals into the environment, and their release will remain a problem that needs to be addressed. Bioremediation technology can fulfil this gap, being a cost-effective technology that can be applied both in situ and ex situ. By using natural autochthonous communities, bioremediation can be applied to restore natural ecosystems like estuarine areas and rivers, and used in WWTPs, can prevent the release of pharmaceuticals into the environment. In the latter, this technology can help to improve biological treatments, maintaining its main goal of removing organic matter and nutrients while exploring and enhancing the bacterial community that can also degrade pharmaceuticals. By analyzing the bacteria present in the biological reactor, it is possible to select those with better skills for the degradation of pharmaceutical compounds, to develop a bacterial cocktail, and use it to increase the biodegradation in the biological reactor without compromising the degradation of the bulk organic matter. However, this can be difficult to develop since different wastewater facilities may have different biological treatments and the associated microbial community can change due to the type of influent, season condition and WWTP configuration. Thus, to obviate this problem, bioremediation could be applied in a tertiary treatment, after the biological treatment, although, a tertiary treatment requires space. Both options present challenges that should be addressed in future studies. Finally, another aspect that should be studied in the future is the nutritional status of the contaminated site. This is a very important issue, since the amount of nutrients in the system is a limiting factor for bacterial growth and the degradation of pharmaceuticals. To avoid eutrophication or nutrient depletion, an optimal C: N: P ratio must be ensured. In the same way, to allow the growth and survival of the bioaugmentation formula in the contaminated environment, a proper amount of nutrients should be available in the matrix. This amount should be directly correlated with the concentration of bacteria to be added to the environment, and in consequence, directly correlated with the concentration of the contaminant (i.e., the amount of carbon source) in the affected site. So, this is also an aspect that should be explored in the development of the bioremediation technology.

Despite the various issues that need further investigation, bioremediation remains a promising solution to prevent pharmaceutical products reaching the environment or to remediate ecosystems impacted by these compounds. In this review, it has been shown that microbial enrichment processes allow the obtaining of bacteria capable of effectively degrading different classes of pharmaceuticals. However, the use of microorganisms added to the affected site can cause negative and undesired impacts or result in low removal efficiencies of the target compound. Introducing exogenous microorganisms into the environment can disrupt and affect the dynamics of the natural community and the functioning of the ecosystem. To overcome this constraint, microbial enrichments should be carried out with autochthonous microorganisms recovered from the affected site. In doing so, the impacts to the natural community can be diminished. Moreover, this microbial community is likely to be better adapted to the contaminated environment and exhibit better performance. As such, the development of bioremediation technologies should take into account the potential of native degrading communities, to ensure a better and more sustainable solution for tackling environmental contamination by pharmaceutical compounds.

## Data Availability

The data presented in this study are available in the current article and the Supplementary Material associated with it, freely available at Toxics.

## Supplementary Materials

The following are available online at https://www.mdpi.com/article/10.3390/toxics9100257/s1, Table S1: Pharmaceutical’s concentration detected in surface water samples (expressed in ng L^−1^), Table S2: Pharmaceutical’s concentration detected in groundwater samples (expressed in ng L^−1^), Table S3: Pharmaceutical’s concentration detected in seawater samples (expressed in ng L^−1^), Table S4: Pharmaceutical’s concentration detected in drinking water samples (expressed in ng L^−1^), Table S5: Pharmaceutical’s concentration detected in sediment and soils samples (expressed in ng g^−1^), Table S6: Pharmaceutical’s concentration detected in sludge samples (expressed in ng g^−1^), Table S7: Pharmaceutical’s concentration detected in wastewaters effluent samples (expressed in ng L^−1^).
