# Supplementary material for: Pharmaceutical Compounds in Aquatic Environments—Occurrence, Fate and Bioremediation Prospective
Source: Toxics. 2021 Oct 9;9(10):257. doi: 10.3390/toxics9100257 (PMC8537644; doi:10.3390/toxics9100257)
Supplement: Supplementary file 1 [file toxics-09-00257-s001.zip › toxics-1352338-sm-final.pdf]

toxics

MDPI

Supplementary Materials: Pharmaceutical Compounds in Aquatic Environments—Occurrence, Fate and Bioremediation Prospective

Joana P. Fernandes, C. Marisa R. Almeida, Maria A. Salgado, Maria F. Carvalho and Ana P. Mucha

Table S1. Pharmaceutical's concentration detected in surface water samples (expressed in ng/L).

Therapeutic Class	Pharmaceutical	Location	Concentration (ngL^{-1})	Reference
Antibiotics	Ciprofloxacin	Spain	34.6	[1]
		Spain	740	[2]
		Spain	23	[3]
		Spain	0.53–20.00	[4]
		Serbia	28.2	[5]
		Spain	34.6	[6]
		Spain	17–540	[7]
	Norfloxacin	Spain	37.2	[1]
		Spain	54	[2]
		Spain	37.2	[6]
		Spain	5–62	[7]
	Ofloxacin	Spain	50.2	[1]
		Spain	400	[2]
		Spain	20–33	[3]
		Spain	0.07–109.50	[4]
		France	3.2	[8]
		Spain	50.2	[6]
	Sulfamethoxazole	Spain	15.6	[1]
		Portugal	9.14–53.3	[9]
		Spain	33	[2]
		USA	66.7	[10]
		Spain	16–79	[3]
		UK	1.8	[11]
		Spain	0.16–41.51	[4]
		France	1.9	[8]
		Spain	15.6	[6]
		Spain	19–227	[7]
		Mexico	76–722	[12]
	Trimethoprim	Spain	3.0	[1]
		Portugal	3.89 to 15.7	[9]
		Spain	151	[2]
		USA	4.1	[10]
Antibiotics	Trimethoprim	Spain	5–9	[3]
		UK	22	[11]
		Spain	0.49–150.43	[4]
		Serbia	8.1	[5]
		France	0.9	[8]
		Spain	3.0	[6]
		Spain	3–2046	[7]
		Mexico	34–120	[12]
	Erythromycin	UK	132–1378	[13]
		Spain	78	[2]
		Spain	0.45–18.58	[4]

		Serbia	292	[5]
		Portugal	38.80	[14]
	Erythromycin–H_2O	USA	12.1	[10]
	Clarithromycin	Spain	91	[2]
		USA	17.6	[10]
		Spain	59	[3]
		UK	43.5	[11]
		Spain	0.09–65.63	[4]
		Serbia	616	[5]
		Portugal	39.10	[14]
	Enrofloxacin	Spain	70	[2]
	Lincomycin	Spain	47	[2]
	Pefloxacin	Spain	64	[2]
	Roxithromycin	Spain	12	[2]
		France	4.9	[8]
	Sarafloxacin	Spain	55	[2]
	Sulfamethizole	USA	15.6	[10]
	Flumequine	Spain	20	[2]
	Azithromycin	Spain	5–41	[3]
		Spain	2.26–33.22	[4]
		Portugal	35.66	[14]
	Cefalexin	Spain	0.40–1.40	[4]
		Serbia	283	[5]
**Antibiotics**	Tetracycline	Spain	5.92–27.40	[4]
	Nalidixic Acid	Spain	14	[2]
	Oxolinic Acid	Spain	23	[2]
	Pipedimic acid	Spain	245	[2]
	Metronidazole	Spain	0.96–65.93	[4]
		France	0.3	[8]
	Moxifloxacin	Spain	205	[2]
	Mefenamic acid	UK	9–97	[13]
	Sulfadiazine	Spain	7–51	[7]
**Nonsteroidal anti-inflammatory drug (NSAIDs)**	Diclofenac	Spain	16.9	[1]
		France	1.36–33.2	[15]
		UK	76–2991	[13]
		Spain	16.9	[6]
		Spain	358	[2]
		Spain	18–52	[3]
		UK	21.5	[11]
		Spain	26.63–280	[4]
		Serbia	324	[5]
		France	5.4	[8]
		Spain	22–650	[7]
		Portugal	51.24	[14]
		Mexico	258–1398	[12]
	Ibuprofen	Spain	59.0	[1]
		France	4.5	[15]
		UK	205–4838	[13]
		Spain	2850	[2]
		USA	37.9	[10]
		Spain	380	[3]
		UK	27.5	[11]
		Spain	3.91–867.82	[4]
		Serbia	346	[5]
		France	5.5	[8]
		Spain	59	[6]

	Ibuprofen	Mexico	184–1106	[12]
	Ketoprofen	France	14.5	[15]
		Spain	70	[2]
		Spain	6.37–356.79	[4]
		Serbia	45	[5]
		France	3.4	[8]
		Spain	4–57	[7]
	Tenoxicam	Spain	0.02–1.59	[4]
	Naproxen	France	9.1	[15]
		Spain	285	[2]
		USA	8.2	[10]
		Spain	7–156	[3]
		UK	127	[11]
**Nonsteroidal anti-inflammatory drug (NSAIDs)**		Spain	12.21–289.47	[4]
		Serbia	74.2	[5]
		France	3.5	[8]
		Mexico	732–4880	[12]
	Piroxicam	Spain	5–11	[3]
		Spain	0.03–5.06	[4]
	Meloxicam	Spain	74	[3]
		Spain	0.01–4.00	[4]
		Serbia	1.8	[5]
	Indomethacin	Spain	11	[3]
		Spain	1.55–137.44	[4]
		Serbia	19.5	[5]
	Indomethacin	Mexico	19–362	[12]
	Phenazone	Spain	12	[3]
		Spain	0.07–40.72	[4]
		Serbia	12.5	[5]
**Antidepressants**	Norfluoxetine	Spain	0.84–3.26	[4]
	Sertraline	Spain	1.06–144.87	[4]
		Portugal	23.30	[14]
	Paroxetine	Spain	40	[3]
**Antidepressants**	Paroxetine	Spain)	0.27–3.41	[4]
	Venlafaxine	Spain	575	[2]
		Spain	43	[3]
		UK	31.1	[11]
		Spain	1.15–127.62	[4]
		Serbia	5.3	[5]
	Citalopram	Spain	9–11	[3]
		Spain	0.08–31.83	[4]
		Portugal	52.97	[14]
	Trazadone	Spain	4	[3]
		Spain	0.04–40.04	[4]
	Fluoxetine	Spain	0.59–17.28	[4]
**Blood lipid lowering agents**	Fenofibrate	Spain	21.4	[6]
	Gemfibrozil	France	2.3	[15]
		USA	38.2	[10]
		Spain	304	[2]
		Spain	9.80–302.67	[4]
		Spain	22–284	[3]
		Portugal	10.34	[14]
		Mexico	9–368	[12]
	Pravastatine	France	1.6	[8]
		Spain	0.53–10.81	[4]
	Bezafibrate	France	3.4	[8]

		UK	42.1	[11]
		Spain	49	[2]
		Spain	0.82–55.64	[4]
		Spain	16	[3]
		Serbia	18.18	[5]
		Spain	8–67	[7]
		Portugal	15.52	[14]
		Mexico	286–2100	[12]
	Atorvastatin	UK	7	[11]
		Spain	42	[2]
**Blood lipid lowering agents**	Atorvastatin	Spain	0.12–8.64	[4]
		Spain	2–3	[3]
		Serbia	9.09	[5]
	Fluvastatin	Spain	0.06–4.19	[4]
	Fenofibric acid	France	0.6	[8]

Table S2. Pharmaceutical's concentration detected in groundwater samples (expressed in ng/L).

Therapeutic Class	Pharmaceutical	Location	Concentration (ngL^{-1})	Reference
Antibiotics	Azithromycin	Spain	30.7–1620	[16]
	Clarithromycin	Spain	2.87–20.5	[16]
	Chlortetracycline	Spain	34.2	[16]
	Ciprofloxacin	Spain	51–443	[16]
	Danofloxacin	Spain	58.7–543	[16]
	Doxycycline	Spain	27.6–188	[16]
	Enoxacin	Spain	69.3–323	[16]
	Enrofloxacin	Spain	65.2–264	[16]
	Erythromycin	Spain	41.3	[16]
	Norfloxacin	Spain	81–462	[16]
	Ofloxacin	Spain	43.3–367	[16]
	Oxytetracycline	Spain	12.2–41	[16]
	Roxithromycin	Spain	3.23–23.8	[16]
		France	1.3	[8]
	Flumequine	Spain	6.05–10.3	[16]
	Josamycin	Spain	3.8	[16]
	Spiramycin	Spain	17.2–2980	[16]
	Sulfadiazine	Spain	37.1–208	[16]
	Sulfamethazine	Spain	29.1–29.2	[16]
	Sulfamethoxazole	Spain	18.2–65	[16]
		France	3.0	[8]
	Tetracycline	Spain	56.3–141	[16]
	Tilmicosin	Spain	5.71–820	[16]
	Trimethoprim	Spain	4.89–9.41	[16]
		France	1.4	[8]
Nonsteroidal anti-inflammatory drug (NSAIDs)	Diclofenac	Spain	1.17–380	[16]
		France	9.7	[8]
	Ibuprofen	Serbia	92	[5]
		Spain	2.12–988	[16]
	Naproxen	Serbia	27.6	[5]
		Spain	5.59	[16]
Nonsteroidal anti-inflammatory drug (NSAIDs)	Naproxen	France	1.2	[8]
	Phenazone	Serbia	23.4	[5]
		Spain	2.13–39.7	[16]
	Ketoprofen	Spain	29.5–215	[16]
		France	[8]	[8]
	Mefenamic acid	Spain	13.5–64.3	[16]

Antidepressants	Fluoxetine	Spain	21	[16]
	Paroxetine	Spain	5.17–30.2	[16]
Blood lipid lowering agents	Fenofibric acid	France	0.4	[8]
	Atorvastatin	Spain	5.12–15.9	[16]
	Bezafibrate	Spain	0.527–25.8	[16]
	Fenofibrate	Spain	22.3–74.2	[16]
	Gemfibrozil	Spain	0.821–751	[16]
	Pravastatin	Spain	12.2	[16]

Table S3. Pharmaceutical's concentration detected in seawater samples (expressed in ng/L).

Therapeutic Class	Pharmaceutical	Location	Concentration (ngL^{-1})	Reference
Antibiotics	Clarithromycin	Spain	17	[3]
	Ofloxacin	Spain	2	[3]
	Sulfamethoxazole	Spain	9	[3]
	Trimethoprim	Spain	1	[3]
Nonsteroidal anti-inflammatory drug (NSAIDs)	Diclofenac	Spain	4	[3]
	Ibuprofen	Spain	16	[3]
	Indomethacin	Spain	3	[3]
	Naproxen	Spain	6	[3]
	Phenazone	Spain	2	[3]
Antidepressants	Citalopram	Spain	4	[3]
	Venlafaxine	Spain	52	[3]
	Trazadone	Spain	1	[3]
Blood lipid lowering agents	Gemfibrozil	Spain	23	[3]
	Atorvastatin	Spain	1	[3]
	Bezafibrate	Spain	2	[3]

Table S4. Pharmaceutical's concentration detected in drinking water samples (expressed in ng/L).

Therapeutic Class	Pharmaceutical	Location	Concentration (ngL^{-1})	Reference
Antibiotics	Sulfamethoxazole	Spain	0.5	[3]
		Portugal	1.3	[17]
		USA	12.7	[18]
		USA	1.3–8.2	[19]
		USA	113	[20]
		Switzerland	15–17	[21]
	Sulfadiazine	Portugal	1	[17]
	Sulfamethazine	Portugal	0.5	[17]
	Sulfapyridine	Portugal	1.9	[17]
	Erythromycin	Portugal	5	[17]
	Erythromycin-H_2O	USA	13.8	[18]
	Clarithromycin	USA	0.2	[18]
	Trimethoprim	USA	19.8	[18]
		USA	1.7–4.7	[19]
		USA	0.7	[20]
		Switzerland	0.4–3	[21]
	Sulfamethizole	USA	1	[20]
	Lincomycin	USA	2.0–4.4	[19]
	Azithromycin	Switzerland	10	[21]
	Norfloxacin	Switzerland	2	[21]
Nonsteroidal anti-inflammatory drug (NSAIDs)	Ibuprofen	USA (raw)	5850	[22]
		USA (finished)	930	
		France	0.6	[15]
		Spain	5	[3]
		Portugal	0.021	[17]
		USA	10.2	[18]

		USA	2.0–72.8	[19]
		Spain	21.24	[6]
	Diclofenac	France	2.5	[15]
		Portugal	11	[17]
		USA	9.4	[18]
Nonsteroidal anti-inflammatory drug (NSAIDs)	Diclofenac	Switzerland	0.7–3	[21]
	Ketoprofen	France	3.0	[15]
		Serbia	16	[5]
		Switzerland	4–8	[21]
	Naproxen	France	0.2	[15]
		Portugal	6	[17]
		USA	5.1	[18]
		Switzerland	4–12	[21]
	Indomethacin	Spain	6	[3]
		Portugal	37	[17]
	Nimesulide	Portugal	27	[17]
Antidepressants	Fluoxetine	USA	19.2	[18]
	Amitryptilline	France	1.4	[15]
Blood lipid lowering agents	Gemfibrozil	USA	1.2	[20]
		Portugal	18	[17]
		Spain	8	[3]
	Atorvastatin	Spain	1	[3]

Table S5. Pharmaceutical's concentration detected in sediment and soils samples (expressed in ng/g).

Therapeutic Class	Pharmaceutical	Location	Concentration (ng g^{-1})	Reference
Antibiotics	Ciprofloxacin	Spain	4.6–7.3	[1]
		Spain	5.95	[6]
		Spain	0.10–3.79	[4]
		China	42	[23]
	Norfloxacin	Spain	6.8–8.4	[1]
		Malaysia	18–96	[24]
		China	17.9	[23]
	Ofloxacin	Spain	8.95–12.03	[6]
		Spain	0.09–2.99	[4]
		Spain	2.7–3.3	[1]
	Sulfamethoxazole	Spain	1.1	[1]
		USA	0.7	[10]
		Spain	0.07–0.26	[4]
		Poland	2.34–419.2	[25]
	Tetracycline	Spain	6.5	[1]
		Spain	5.92	[4]
	Trimethoprim	Spain	0.2–1.6	[1]
		Spain	0.02–0.25	[26]
		USA	18.2	[10]
		Spain	0.03–0.19	[4]
		Poland	1.74–2.46	[25]
		Malaysia	3–60	[24]
	Sulfamethoxypyridazine	Spain	0.11–0.37	[26]
	Sulfamethazine	Spain	0.24–1.15	[26]
		Poland	1.76	[25]
	Erithromycin	Spain	1.13	[4]
	Erithromycin-H_2O	USA	3.4	[10]
	Azithromycin	Spain	23.92	[4]
	Clarithromycin	Spain	12.72	[4]
	Cefalexin	Spain	0.40	[4]

	Metronidazole	Spain	0.12–12.61	[4]
	Sulfamethiazole	Poland	12.85–20.84	[25]
Antibiotics	Doxycycline	Malaysia	63–728	[24]
	Enrofloxacin	Malaysia	36–378	[24]
		China	24.4	[23]
		Brazil	26.69	[27]
	Flumequine	Malaysia	8–1331	[24]
	Tylosin	Malaysia	6–679	[24]
	Lomefloxacin	China	11	[23]
	Sulfachloropyridazine	Poland	0.47–1.07	[25]
	Sulfathiazole	Poland	1.77	[25]
	Chloramphenicol	Spain	0.17–2.10	[26]
Nonsteroidal anti-inflammatory drug (NSAIDs)	Diclofenac	Spain	0.81–15.39	[26]
		Spain	1.29–4.14	[4]
		Poland	2.1	[28]
	Ibuprofen	Spain	0.91–24.93	[26]
		Spain	12.56	[4]
		Poland	1.0–8.0	[28]
	Indomethacin	Spain	0.47–8.99	[26]
		Spain	0.47–2.94	[4]
	Phenazone	Spain	0..06	[4]
	Ketoprofen	Spain	5.79–12.54	[4]
	Naproxen	Spain	0.82–3.38	[4]
	Piroxicam	Spain	0.15	[4]
	Meloxicam	Spain	0.08	[4]
	Tenoxicam	Spain	0.66	[4]
	Flurbiprofen	Poland	6.5–8.8	[28]
	Mefenamic acid	Spain	0.18–3.34	[26]
Antidepressants	Fluoxetine	Spain	0.34	[4]
	Norfluoxetine	Spain	0.14–0.60	[4]
	Paroxetine	Spain	0.05–0.76	[4]
	Sertraline	Spain	1.15– 119.28	[4]
	Citalopram	Spain	0.23–7.79	[4]
	Venlafaxine	Spain	0.05–1.94	[4]
Antidepressants	Trazodone	Spain	0.09–8.08	[4]
Blood lipid lowering agents	Bezafibrate	Spain	0.09–0.41	[4]
	Gemfibrozil	Spain	0.16–1.92	[4]
		Spain	0.08–0.39	[26]
	Atorvastatin	Spain	0.03–0.65	[4]
	Pravastatin	Spain	0.30	[4]
	Fluvastatin	Spain	0.22–4.53	[4]
	Fenofibrate	Spain	16.1	[1]
		Spain	13.20–17.23	[6]

Table S6. Pharmaceutical's concentration detected in sludge samples (expressed in ng/g).

Therapeutic Class	Pharmaceutical	Location	Concentration (ng g^{-1})	Reference
Antibiotics	Trimethoprim	UK	21.5	[11]
		Malaysia	9–3412	[24]
	Doxycycline	Malaysia	309–78516	[24]
		China	1050–10910	[29]
	Enrofloxacin	Malaysia	112–26863	[24]
		Brazil	30970	[27]
		China	33260–1420760	[29]
	Erythromycin	Malaysia	12–32	[24]
	Norfloxacin	Malaysia	31–1886	[24]
		Brazil	4550	[27]
		China	2760–225450	[29]
	Sulfadiazine	Malaysia	12–5773	[24]
		China	800–3120	[27]
	Tilmicosin	Malaysia	13–85	[24]
	Tylosin	Malaysia	100–13740	[24]
	Ciprofloxacin	Brazil	2130	[27]
		China	29590–45590	[29]
	Fleroxacin	China	2220–99430	[29]
	Flumequine	Malaysia	21–51912	[24]
	Sulfamethoxazole	China	840–2800	[29]
	Oxytetracycline	China	10560–59590	[29]
	Chlortetracycline	China	17680–27590	[29]
	Methacycline	China	960–5860	[29]
	Lomefloxacin	China	5530–44160	[29]
	Danofloxacin	China	2480–3060	[29]
	Sulfanilamide	China	40–1590	[29]
	Sulfamerazine	China	90–660	[29]
	Sulfadimidine	China	180–6040	[29]
	Difloxacin	China	2630–12380	[29]
	Sulfamonomethoxine	China	60–4080	[29]
	Sulfaguanidine	China	250–1550	[29]
	Sulfachloropyridazine	China	360–3510	[29]
Nonsteroidal anti-inflammatory drug (NSAIDs)	Ibuprofen	UK	174	[11]
		Poland	96	[28]
	Diclofenac	UK	23.5	[11]
		Poland	20	[28]
	Naproxen	UK	39.8	[11]
		Poland	10	[28]
	Flurbiprofen	Poland	98	[28]
Antidepressants	Citalopram	UK	657	[11]
	Venlafaxine	UK	37.9	[11]
	Fluoxetine	UK	188	[11]
	Sertraline	UK	1138	[11]
	Norfluoxetine	UK	124	[11]
	Mirtazapine	UK	66.1	[11]

Table S7. Pharmaceutical's concentration detected in wastewaters effluent samples (expressed in ng/L).

Therapeutic Class	Pharmaceutical	Location	Concentration (ng L^{-1})	Reference
Antibiotics	Erythromycin	UK	466–1857	[13]
		Spain	82	[2]
		Spain	14–17	[3]
		China	42–244.0	[30]
	Ciprofloxacin	Spain	2292	[2]
		Spain	104–245	[3]
		USA	2200	[31]
		Serbia	278	[5]
		Spain	3194–4719	[7]
	Clarithromycin	Spain	247	[2]
		Spain	19–192	[3]
		UK	1065	[11]
		USA	8100	[31]
	Enrofloxacin	Spain	220	[2]
		USA	34	[31]
	Flumequine	Spain	41	[2]
		USA	15.6	[31]
	Lincomycin	Spain	142	[2]
		USA	32	[31]
	Nalidixic acid	Spain	60	[2]
	Moxifloxacin	Spain	540	[2]
		China	5.1–7.7	[30]
	Azithromycin	Spain	31–170	[3]
		UK	87.2	[11]
		China	20.7–446.5	[30]
		USA	1300	[31]
	Ofloxacin	Spain	157–191	[3]
		China	26.7–310.0	[30]
		USA	2100	[31]
		Serbia	220	[5]
		Spain	925	[2]
	Sulfamethoxazole	Spain	222	[3]
Antibiotics	Sulfamethoxazole	UK	47.5	[11]
		China	44.5–1296.3	[30]
		USA	7400	[31]
		Serbia	432	[5]
		Spain	432	[2]
		Spain	190–8963	[7]
		Mexico	440–1215	[12]
	Trimethoprim	Spain	10–100	[3]
		UK	769	[11]
		China	6.7–188.0	[30]
		USA	570	[31]
		Serbia	259	[5]
		Spain	232	[2]
		Spain	61–5843	[7]
		Mexico	130–395	[12]
	Sulfamerazine	USA	30	[31]
	Sulfamethizole	USA	60	[31]
	Cefalexin	Serbia	803	[5]
	Ampicillin	USA	160	[31]
	Penicillin G	USA	30	[31]
	Penicillin V	USA	86	[31]

	Sarafloxacin	USA	130	[31]
		Spain	52	[2]
	Sulfadiazine	China	14–189.7	[30]
		USA	20	[31]
		Spain	24–5477	[7]
	Sulfamethazine	China	2–48.6	[30]
		USA	40	[31]
		Spain	11	[2]
	Sulfadimethoxine	USA	52	[31]
	Sulfachloropyridazine	USA	30	[31]
	Pefloxacin	Spain	122	[2]
	Norfloxacin	China	9.4–134.3	[30]
		USA	140	[31]
Antibiotics	Norfloxacin	Spain	310	[2]
		Spain	40–463	[7]
	Roxithromycin	China	36.5–512.4	[30]
		USA	120	[31]
		Spain	18	[2]
	Sulfathiazole	USA	92	[31]
		Spain	30	[2]
	Pipedimic acid	Spain	430	[2]
	Mefenamic acid	UK	15–108	[13]
	Metronidazole	Spain	121	[3]
Nonsteroidal anti-inflammatory drug (NSAIDs)	Diclofenac	France	210.7–486.4	[15]
		UK	401–2830	[13]
		Spain	690	[2]
		Spain	184–376	[3]
		UK	436	[11]
		China	1.0–4.7	[30]
		Serbia	1338	[5]
		Mexico	466–2180	[12]
		Spain	683–1932	[7]
	Ibuprofen	France	17.7–219.0	[15]
		UK	863–4617	[13]
		Spain	15100	[2]
		UK	1290	[11]
		China	3.5–41.6.	[30]
		USA	4500	[31]
		Serbia	20130	[5]
	Ketoprofen	France	21.8–1080.6	[15]
		Spain	583	[2]
		Spain	39–560	[3]
		Serbia	247	[5]
		Spain	13–374	[7]
	Naproxen	France	42.1–289.1	[15]
		Spain	710	[2]
Nonsteroidal anti-inflammatory drug (NSAIDs)	Naproxen	Spain	97–150	[3]
		UK	3516	[11]
		USA	3000	[31]
		Serbia	208	[5]
		China	6.7–7.7	[30]
		Mexico	49–392	[12]
	Phenazone	Spain	9–49	[3]
		Serbia	13.5	[5]
	Piroxicam	Spain	87	[3]
	Meloxicam	Spain	735	[3]

		Serbia	5.0	[5]
	Tenoxicam	Spain	19	[3]
	Indomethacin	Mexico	38–305	[12]
Antidepressants	Citalopram	Spain	49–288	[3]
		UK	323	[11]
		China	2–162	[32]
	Venlafaxine	Spain	364–376	[3]
		UK	355	[11]
		Serbia	154	[5]
		Spain	875	[2]
	Trazodone	Spain	29	[3]
	Amitryptiline	France	6.0	[15]
	Fluoxetine	Spain	28	[3]
		UK	26.5	[11]
		China	10	[32]
		USA	50	[31]
	Paroxetine	Spain	386	[3]
	Sertraline	UK	47	[11]
		China	9–59	[32]
	Mirtazapine	UK	55	[11]
	Chlorimipramine	China	4–35	[32]
	Norfluoxetine	UK	30	[11]
Blood lipid lowering agents	Gemfibrozil	France	13.3–17.2	[15]
		USA	190	[31]
		China	0.6–10.2	[30]
		Spain	2008	[2]
		Spain	178–1018	[3]
		Mexico	20–380	[12]
	Bezafibrate	China	2.7–128.1	[30]
		UK	892	[11]
		Spain	312	[2]
		Spain	7–26	[3]
		Spain	40–132	[7]
		Mexico	265–950	[12]
	Atorvastatin	China	0.7–0.8	[30]
		UK	60.5	[11]
		Spain	209	[2]
		Spain	27–111	[3]
		Serbia	40.5	[5]
		Canada	10–122	[33]
	Pravastatin	Spain	69	[2]
		Spain	36	[3]
	Fluvastatin	Spain	12	[3]
	Simvastatin	China	8.4–129	[30]
	Rosuvastatin	Canada	190–552	[33]

References

1. Vazquez-Roig, P.; Andreu, V.; Blasco, C.; Picó, Y. Risk assessment on the presence of pharmaceuticals in sediments, soils and waters of the Pego–Oliva Marshlands (Valencia, eastern Spain). *Sci. Total Environ*. **2012**, *440*, 24–32, doi:10.1016/j.scitotenv.2012.08.036.
2. Gracia-Lor, E.; Sancho, J. V.; Hernández, F. Multi-class determination of around 50 pharmaceuticals, including 26 antibiotics, in environmental and wastewater samples by ultra-high performance liquid chromatography–tandem mass spectrometry. *J. Chromatogr. A* **2011**, *1218*, 2264–2275, doi:10.1016/j.chroma.2011.02.026.

3. Gros, M.; Rodríguez-Mozaz, S.; Barceló, D. Fast and comprehensive multi-residue analysis of a broad range of human and veterinary pharmaceuticals and some of their metabolites in surface and treated waters by ultra-high-performance liquid chromatography coupled to quadrupole-linear ion trap tandem. *J. Chromatogr. A* **2012**, *1248*, 104–121, doi:10.1016/j.chroma.2012.05.084.

4. Osorio, V.; Larrañaga, A.; Aceña, J.; Pérez, S.; Barceló, D. Concentration and risk of pharmaceuticals in freshwater systems are related to the population density and the livestock units in Iberian Rivers. *Sci. Total Environ.* **2016**, *540*, 267–277, doi:10.1016/j.scitotenv.2015.06.143.

5. Petrović, M.; Škrbić, B.; Živančev, J.; Ferrando-Climent, L.; Barcelo, D. Determination of 81 pharmaceutical drugs by high performance liquid chromatography coupled to mass spectrometry with hybrid triple quadrupole–linear ion trap in different types of water in Serbia. *Sci. Total Environ.* **2014**, *468*, 415–428, doi:10.1016/j.scitotenv.2013.08.079.

6. Vazquez-Roig, P.; Segarra, R.; Blasco, C.; Andreu, V.; Picó, Y. Determination of pharmaceuticals in soils and sediments by pressurized liquid extraction and liquid chromatography tandem mass spectrometry. *J. Chromatogr. A* **2010**, *1217*, 2471–2483, doi:10.1016/j.chroma.2009.11.033.

7. Mijangos, L.; Ziarrusta, H.; Ros, O.; Kortazar, L.; Fernández, L.A.; Olivares, M.; Zuloaga, O.; Prieto, A.; Etxebarria, N. Occurrence of emerging pollutants in estuaries of the Basque Country: Analysis of sources and distribution, and assessment of the environmental risk. *Water Res.* **2018**, *147*, 152–163, doi:10.1016/j.watres.2018.09.033.

8. Vulliet, E.; Cren-Olivé, C. Screening of pharmaceuticals and hormones at the regional scale, in surface and groundwaters intended to human consumption. *Environ. Pollut.* **2011**, *159*, 2929–2934, doi:10.1016/j.envpol.2011.04.033.

9. Madureira, T.V.; Barreiro, J.C.; Rocha, M.J.; Rocha, E.; Cass, Q.B.; Tiritan, M.E. Spatiotemporal distribution of pharmaceuticals in the Douro River estuary (Portugal). *Sci. Total Environ.* **2010**, *408*, 5513–5520, doi:10.1016/j.scitotenv.2010.07.069.

10. Klosterhaus, S.L.; Grace, R.; Hamilton, M.C.; Yee, D. Method validation and reconnaissance of pharmaceuticals, personal care products, and alkylphenols in surface waters, sediments, and mussels in an urban estuary. *Environ. Int.* **2013**, *54*, 92–99, doi:10.1016/j.envint.2013.01.009.

11. Petrie, B.; Youdan, J.; Barden, R.; Kasprzyk-Hordern, B. Multi-residue analysis of 90 emerging contaminants in liquid and solid environmental matrices by ultra-high-performance liquid chromatography tandem mass spectrometry. *J. Chromatogr. A* **2016**, *1431*, 64–78, doi:10.1016/j.chroma.2015.12.036.

12. Rivera-Jaimes, J.A.; Postigo, C.; Melgoza-Alemán, R.M.; Aceña, J.; Barceló, D.; López de Alda, M. Study of pharmaceuticals in surface and wastewater from Cuernavaca, Morelos, Mexico: Occurrence and environmental risk assessment. *Sci. Total Environ.* **2018**, *613*, 1263–1274, doi:10.1016/j.scitotenv.2017.09.134.

13. Kay, P.; Hughes, S.R.; Ault, J.R.; Ashcroft, A.E.; Brown, L.E. Widespread, routine occurrence of pharmaceuticals in sewage effluent, combined sewer overflows and receiving waters. *Environ. Pollut.* **2017**, *220*, 1447–1455, doi:10.1016/j.envpol.2016.10.087.

14. Pereira, A.M.P.T.; Silva, L.J.G.; Laranjeiro, C.S.M.; Meisel, L.M.; Lino, C.M.; Pena, A. Human pharmaceuticals in Portuguese rivers: The impact of water scarcity in the environmental risk. *Sci. Total Environ.* **2017**, *609*, 1182–1191, doi:10.1016/j.scitotenv.2017.07.200.

15. Togola, A.; Budzinski, H. Multi-residue analysis of pharmaceutical compounds in aqueous samples. *J. Chromatogr. A* **2008**, *1177*, 150–158, doi:10.1016/j.chroma.2007.10.105.

16. López-Serna, R.; Jurado, A.; Vázquez-Suñé, E.; Carrera, J.; Petrović, M.; Barceló, D. Occurrence of 95 pharmaceuticals and transformation products in urban groundwaters underlying the metropolis of Barcelona, Spain. *Environ. Pollut.* **2013**, *174*, 305–315, doi:10.1016/j.envpol.2012.11.022.

17. Gaffney, V.J.; Almeida, C.M.M.; Rodrigues, A.; Ferreira, E.; Benoliel, M.J.; Cardoso, V.V. Occurrence of pharmaceuticals in a water supply system and related human health risk assessment. *Water Res*. **2015**, *72*, 199–208, doi:10.1016/j.watres.2014.10.027.

18. Padhye, L.P.; Yao, H.; Kung'u, F.T.; Huang, C.-H. Year-long evaluation on the occurrence and fate of pharmaceuticals, personal care products, and endocrine disrupting chemicals in an urban drinking water treatment plant. *Water Res*. **2014**, *51*, 266–276, doi:10.1016/j.watres.2013.10.070.

19. Wang, C.; Shi, H.; Adams, C.D.; Gamagedara, S.; Stayton, I.; Timmons, T.; Ma, Y. Investigation of pharmaceuticals in Missouri natural and drinking water using high performance liquid chromatography-tandem mass spectrometry. *Water Res*. **2011**, *45*, 1818–1828, doi:10.1016/j.watres.2010.11.043.

20. Schaider, L.A.; Rudel, R.A.; Ackerman, J.M.; Dunagan, S.C.; Brody, J.G. Pharmaceuticals, perfluorosurfactants, and other organic wastewater compounds in public drinking water wells in a shallow sand and gravel aquifer. *Sci. Total Environ*. **2014**, *468*, 384–393, doi:10.1016/j.scitotenv.2013.08.067.

21. Morasch, B. Occurrence and dynamics of micropollutants in a karst aquifer. *Environ. Pollut.* **2013**, *173*, 133–137, doi:10.1016/j.envpol.2012.10.014.

22. Loraine, G.A.; Pettigrove, M.E. Seasonal Variations in Concentrations of Pharmaceuticals and Personal Care Products in Drinking Water and Reclaimed Wastewater in Southern California. *Environ. Sci. Technol*. **2006**, *40*, 687–695, doi:10.1021/es051380x.

23. Wu, X.-L.; Xiang, L.; Yan, Q.-Y.; Jiang, Y.-N.; Li, Y.-W.; Huang, X.-P.; Li, H.; Cai, Q.-Y.; Mo, C.-H. Distribution and risk assessment of quinolone antibiotics in the soils from organic vegetable farms of a subtropical city, Southern China. *Sci. Total Environ.* **2014**, *487*, 399–406, doi:10.1016/j.scitotenv.2014.04.015.

24. Ho, Y. Bin; Zakaria, M.P.; Latif, P.A.; Saari, N. Occurrence of veterinary antibiotics and progesterone in broiler manure and agricultural soil in Malaysia. *Sci. Total Environ*. **2014**, *488*, 261–267, doi:10.1016/j.scitotenv.2014.04.109.

25. Siedlewicz, G.; Borecka, M.; Białk-Bielińska, A.; Sikora, K.; Stepnowski, P.; Pazdro, K. Determination of antibiotic residues in southern Baltic Sea sediments using tandem solid-phase extraction and liquid chromatography coupled with tandem mass spectrometry. *Oceanologia* **2016**, *58*, 221–234, doi:10.1016/j.oceano.2016.04.005.

26. Biel-Maeso, M.; Corada-Fernández, C.; Lara-Martín, P.A. Determining the distribution of pharmaceutically active compounds (PhACs) in soils and sediments by pressurized hot water extraction (PHWE). *Chemosphere* **2017**, *185*, 1001–1010, doi:10.1016/j.chemosphere.2017.07.094.

27. Leal, R.M.P.; Figueira, R.F.; Tornisielo, V.L.; Regitano, J.B. Occurrence and sorption of fluoroquinolones in poultry litters and soils from São Paulo State, Brazil. *Sci. Total Environ*. **2012**, *432*, 344–349, doi:10.1016/j.scitotenv.2012.06.002.

28. Kumirska, J.; Migowska, N.; Caban, M.; Łukaszewicz, P.; Stepnowski, P. Simultaneous determination of non-steroidal anti-inflammatory drugs and oestrogenic hormones in environmental solid samples. *Sci. Total Environ*. **2015**, *508*, 498–505, doi:10.1016/j.scitotenv.2014.12.020.

29. Zhao, L.; Dong, Y.H.; Wang, H. Residues of veterinary antibiotics in manures from feedlot livestock in eight provinces of China. *Sci. Total Environ*. **2010**, *408*, 1069–1075, doi:10.1016/j.scitotenv.2009.11.014.

30. Yan, Q.; Gao, X.; Chen, Y.-P.; Peng, X.-Y.; Zhang, Y.-X.; Gan, X.-M.; Zi, C.-F.; Guo, J.-S. Occurrence, fate and ecotoxicological assessment of pharmaceutically active compounds in wastewater and sludge from wastewater treatment plants in Chongqing, the Three Gorges Reservoir Area. *Sci. Total Environ*. **2014**, *470*, 618–630, doi:10.1016/j.scitotenv.2013.09.032.

31. Blair, B.; Nikolaus, A.; Hedman, C.; Klaper, R.; Grundl, T. Evaluating the degradation, sorption, and negative mass balances of pharmaceuticals and personal care products during wastewater treatment. *Chemosphere* **2015**, *134*, 395–401, doi:10.1016/j.chemosphere.2015.04.078.

32. Yuan, S.; Jiang, X.; Xia, X.; Zhang, H.; Zheng, S. Detection, occurrence and fate of 22 psychiatric pharmaceuticals in psychiatric hospital and municipal wastewater treatment plants in Beijing, China. *Chemosphere* **2013**, *90*, 2520–2525, doi:10.1016/j.chemosphere.2012.10.089.
33. Lee, H.-B.; Peart, T.E.; Lewina Svoboda, M.; Backus, S. Occurrence and fate of rosuvastatin, rosuvastatin lactone, and atorvastatin in Canadian sewage and surface water samples. *Chemosphere* **2009**, *77*, 1285–1291, doi:10.1016/j.chemosphere.2009.09.068.
